# Honduras HIV cohort: HLA class I and CCR5-Δ32 profiles and their associations with HIV disease outcome

**DOI:** 10.1128/spectrum.01613-23

**Published:** 2023-11-14

**Authors:** Humberto Valenzuela-Ponce, Candy Carbajal, Maribel Soto-Nava, Daniela Tapia-Trejo, Claudia García-Morales, Wendy Murillo, Ivette Lorenzana, Gustavo Reyes-Terán, Santiago Ávila-Ríos

**Affiliations:** 1 CIENI Centro de Investigación en Enfermedades Respiratorias, Instituto Nacional de Enfermedades Respiratorias Ismael Cosío Villegas, Mexico City, Mexico; 2 Universidad Nacional Autónoma de Honduras, Tegucigalpa, Honduras; 3 Comisión Coordinadora de Institutos Nacional de Salud y Hospitales de Alta Especialidad, Secretar ´ıa de Salud, Mexico City, Mexico; Barnard College, Columbia University, New York, New York, USA

**Keywords:** HLA allele frequencies, HLA haplotype structures, HLA linkage disequilibrium, HLA-HIV disease associations, Honduran Mestizos, Latin America, Mesoamerican Mestizos, HIV disease outcome

## Abstract

Human leukocyte antigen (HLA) polymorphisms represent the strongest genetic modifier of HIV disease progression. Diverse HLA distributions can lead to distinct HIV control landscapes at the population level. We aimed to describe HLA allele and haplotype frequencies (linkage disequilibrium, LD), CCR5-Δ32 frequency, and the impact of these variants on HIV disease outcome. HLA class I loci were typed at 4-digit resolution, and CCR5 variants were determined in 402 HIV clade B-infected, antiretroviral treatment-naïve individuals from Honduras. HLA LD was assessed using Fisher’s exact test. Using univariable and multivariable analyses, we evaluated HLA associations with HIV plasma viral load (pVL) and CD4 counts. We did not find any effect on HIV control between CCR5 genotypes. The previously defined HLA associations were found to be B*57:01/03, B*42:01, A*25:01, and C*12:03 (protective) and B*53:01 and A*68:01 (risk). Consistent with our previous research in a Mesoamerican HIV cohort, Amerindian B*35:12 was associated with poor HIV control. Other HLA-HIV associations not previously described were C*03:04 and B*08:01, which were associated with higher pVL. Overall, this first report highlights the immunogenetic uniqueness of the Honduras population that expresses Amerindian, Caucasian, and African HLA subtypes. These findings not only support this cohort as ideal for identifying HLA correlates of HIV control but also may improve future research regarding allotransplantation and disease association.

**IMPORTANCE:**

We identify both canonical and novel human leukocyte antigen (HLA)-HIV associations, providing a first step toward improved understanding of HIV immune control among the understudied Honduras Mestizo population. Our results are relevant to understanding the protective or detrimental effects of HLA subtypes in Latin America because their unique HLA diversity poses challenges for designing vaccines against HIV and interpreting results from such vaccine trials. Likewise, the description of the HLA profile in an understudied population that shows a unique HLA immunogenetic background is not only relevant for HIV immunology but also relevant in population genetics, molecular anthropology, susceptibility to other infections, autoimmune diseases, and allograft transplantation.

## INTRODUCTION

The human leukocyte antigen (HLA) class I (cI) loci that are encoded within the major histocompatibility complex (MHC) are the most polymorphic genes in the human genome (1). According to the IPD-IMGT/HLA Database (release 3.50, www.ebi.ac.uk/ipd/imgt/hla/), more than 25 thousand alleles that encode for more than 14 thousand distinct proteins have been discovered (2). The HLA cI (*HLA-A*, *HLA-B*, and *HLA-C*) molecules play a crucial role in the ability of the immune system to recognize and develop immunity against viruses, where immunogenic viral peptides (i.e., epitopes) are presented upon HLA proteins on the surface of nucleated cells to be recognized by cytotoxic CD8^+^ T lymphocytes (CTLs) (1). HLA cI polymorphisms represent the strongest genetic modifier of HIV disease progression (3 – 6), and diverse HLA expression distribution can lead to distinct HIV control landscapes at the population level (7 – 19). As portrayed previously, Mesoamerican populations (i.e., Mexico and Central America countries) exhibit extensive HLA diversity that comprises Amerindian-specific subtypes (e.g., B*35:12/14/16/20/43 and B*39:02/05/06/08, among others) (20), which are either very rare or are absent elsewhere in the world (21) and have not been studied extensively in the context of HIV. Understanding the protective or detrimental effects of HLA subtypes in Mestizos from Latin America is critical because the unique HLA diversity poses challenges for designing vaccines against HIV and interpreting results from such vaccine trials.

Likewise, the description of the HLA profile [i.e., allelic frequencies and the patterns of linkage disequilibrium (LD, i.e., the non-random association of subtypes of discrete loci)] in an unstudied population that shows a unique HLA immunogenetic background is not only relevant for HIV immunology (i.e., disentangling HLA-HIV associations in haplotype structures) but also relevant in population genetics, molecular anthropology, susceptibility and resistance to other infectious diseases, autoimmune diseases, and allograft (i.e., organs or tissues) transplantation (22).

An additional investigated genetic factor correlated with HIV acquisition and disease progression is the presence of a 32-base pair deletion in the human CC-type chemokine receptor 5 gene (CCR5-Δ32), i.e., in the early phases of infection, the key co-receptor used by macrophage-tropic strains of HIV to infect peripheral blood mononuclear cells (23 – 25). This deletion leads to the synthesis of a truncated protein that does not allow the proper interaction between HIV particles and the cell surface, thus preventing virion endocytosis (26, 27). CCR5-Δ32 is mainly found in European populations and shows a frequency decrease from Northern to Southeastern Eurasia, with no or rare occurrences in Asians and native populations from Africa, the Americas, and Oceania (28 – 32).

Honduras spans about 112,492 km^2^ and has a population exceeding 9.6 million inhabitants. Spanish is the official language, but indigenous dialects are also spoken in some communities (e.g., Arawakan, Misumalpan, Chibchan, Jicaquean, and Mayan) (33). The Honduran population has a multi-ethnic background, with Mestizos being the majority (85.6%), but also indigenous people (7.7%, e.g., Lencas, Misquitos, Tolupanes, Chortis, Pech, and Tawahkas), English-speaking Garifunas and Afro-descendants (3.3%, descendants of West African, Central African, Island Carib, and Arawakan people), European descendants (1.0%), and Asian descendants (0.7%, including Jews and Arabs) (34 – 36). It is projected that 22,000 (20,000–25,000) adults and children live with HIV in Honduras, with a prevalence rate of 0.2 (0.2–0.3) in adults aged 15 to 49 (37). The HIV epidemic in Honduras is concentrated among men who have sex with men (MSM), female sex workers, and Garifunas. Nearly 60% of infected adults are male, with a high proportion of heterosexual transmissions (38).

Here, we aimed to describe second-field resolution HLA class I allele frequencies (AFs) and haplotype structures (i.e., linkage disequilibrium), the frequency of the CCR5Δ32 variant, and the impact of these variants on HIV disease outcome in a cohort of HIV-1 B clade-infected, antiretroviral treatment (ART)-naïve individuals from Honduras.

## MATERIALS AND METHODS

### Honduras HIV cohort

The source population for this cross-sectional analysis was 402 HIV-1 clade B-infected, ART-naïve non-related individuals recruited from five of the major HIV reference centers in Honduras between March 2013 and June 2015, encompassing University School Hospital (Tegucigalpa, *n* = 38), National Cardio-Pulmonary Institute (Tegucigalpa, *n* = 83), Mario Catarino Rivas Hospital (San Pedro Sula, *n* = 136), Atlántida Hospital (La Ceiba, *n* = 86), and South Hospital (Choluteca, *n* = 59). Participants were recruited at the time of HIV diagnosis at participating reference centers or at follow-up visits prior to initiating ART. No exclusion criteria were applied except for self-declared previous exposure to ART. Demographic data were obtained via questionnaire at the instance of blood sample donation and collected in EDTA Vacutainer tubes (BD, San Jose, CA, USA) for molecular experiments and Cyto-Chex BCT tubes (Streck, Omaha, NE, USA) for CD4^+^ T cell counts (CD4 counts). We collected data on gender, age, HIV risk factor (probable route of infection, self-declared as heterosexual, MSM or bisexual, users of injected drugs, or mother-to-child vertical transmission), marital status, employment status, and years of education. Blood samples, signed consent forms, and questionnaires were sent to the Centre for Research in Infectious Diseases (CIENI) of INER in Mexico City, a World Health Organization-accredited laboratory for HIV genotyping, within the following 48 h of collection. As a participant benefit, HIV clinical data obtained from samples [baseline ART resistance test, plasma viral load (pVL), and CD4 counts] were sent back to HIV reference centers for individuals’ clinical follow-up.

### HIV subtyping, clinical parameters, and assesment of recent infection

The HIV clade was determined using REGA HIV-1 subtyping Tool V3 (39) and confirmed with the Recombination Identification Program (40) using available plasma HIV pol (protease and reverse transcriptase) sequences, obtained and described elsewhere (41). All non-subtype B-infected subjects were removed prior to analysis.

HIV pVL was determined by automated real-time polymerase chain reaction (PCR) using the m2000 system (Abbott, Abbot Park, IL, USA) with a detection limit of 40 HIV RNA copies/mL. CD4^+^ T cell counts were obtained by flow cytometry using the AQUIOS Tetra-1 Panel in AQUIOS CL (Beckman Coulter Life Sciences, Indianapolis, IN, USA).

Recent infected individuals were identified using a previously described multi-assay algorithm, including an incidence test that minimizes false results (42). As described elsewhere (41), the BED HIV-1 Incidence EIA (Sedia, Portland, OR, USA) was applied to all individuals with CD4 counts greater than 200 cells/µL and less than 1 year of HIV diagnosis. Samples with normalized optical density ≤1.5 and pVL <1,000 HIV RNA copies/mL were considered recent seroconversion cases using HIV-1 LAg-Avidity EIA.

### Genomic DNA extraction

Buffy coats (i.e., enriched leukocyte fraction by centrifugation) were isolated from blood samples and cryopreserved (−80°C) until use. Genomic DNA was extracted from buffy coats (200 µL) using the QIAmp Blood Mini Kit (QIAGEN, Valencia, CA, USA), according to the manufacturer’s instructions. DNA specimens’ quality and quantification were assessed using a NanoDrop One spectrophotometer (ThermoFisher Scientific).

### CCR5Δ32 genotyping

As described previously (27), a PCR using a primer pair that contains the deletion of 32 bp of CCR5 was performed (forward: 5′ CTTCATTACACCTGCAGCT 3′ and reverse: 5′ TGAAGATAAGCCTCACAGCC 3′). Two PCR fragments were obtained, comprising CCR5-wt (not truncated or wild type) and CCR5-Δ32 variants (196 and 164 bp, respectively), that were corroborated by electrophoresis on a 2% agarose gel. All the PCR reactions underwent an internal amplification control to be validated. Significance testing of difference proportions was done between Honduras cities using Fisher’s exact test.

### HLA class I typing

HLA class I loci (*HLA-A*, *HLA-C*, and *HLA-B*) were resolved at second field resolution (i.e., subtype or protein level) using a previously described protocol (20, 43). Briefly, a nested PCR with locus-specific primers was used to amplify an ~1,000 bp region spanning exons 2 and 3. These PCR products were directly sequenced using a set of sequencing primers as previously described (44) on a 3730xl Genetic Analyzer (Applied Biosystems, Foster City, USA). HLA subtypes were assigned using UType v7.1 RUO (Applied Biosystems) using the IPD-IMGT/HLA Database (release 3.31.0, January 2018). Primer (allelic) and phase resolution ambiguities were resolved as previously explained (20). As previously defined (43), our HLA typing methodology was validated to be 99.9% accurate in the Latin American Mestizo population by comparing assigned HLA subtypes to those obtained through amplification of exons 1 to 8 (for *HLA-A* and *-C*) and exons 1 to 7 (for *HLA-B*), followed by next generation sequencing in an independent cohort (*n* = 323).

### HLA statistics, linkage disequilibrium, and AF comparison between cities

AFs (2*n*) were calculated by direct gene count. Hardy-Weinberg Equilibrium was assessed using Arlequin (version 3.5.2.2) software (45). LD between HLA allele pairs was assessed using Fisher’s exact tests with multiple comparisons addressed via false discovery rate (*q*-value). For two-loci LD analysis, 6,117 distinct two-way tests were performed (*P* < 0.05 and *q* <0.2 were considered significant). For each of the statistically significant HLA pairs discovered in the two-loci LD analysis, a Fisher’s test was performed against a third locus (3,032 distinct two-way tests; *P* < 0.05 and *q* <0.2 were considered significant). Tests were undertaken in R version 4.1.2 (1 November 2021) (46). To further validate the three-loci HLA haplotype structures, we estimated three-loci HF using the Expectation-Maximization (EM) algorithm in Arlequin v3.5 (45) and compared HF to those obtained by Fisher’s exact tests. Frequency and haplotype plots were prepared using GraphPad Prism v8.3.1 for macOS (GraphPad Software, San Diego, CA, USA). The high-dimensional visualization tool Disentangler (47) was used to plot HLA haplotype structures. All HLA subtypes with AF >0.005 in at least one city were compared using Fisher’s exact tests (*P* < 0.05 was considered significant).

### Most probable ancestry of HLA subtypes and three-loci haplotype structures

In order to work with the maximum integrated data, the most probable ancestry (MPA, i.e., putative origin) for only the three-loci haplotype structures was determined based on the top relative frequencies when investigated in the Allele Frequencies net database (48), where haplotypes were categorized as putative Caucasian (i.e., European), Amerindian (i.e., American Native), African, Oriental, or mixed origin (haplotypes non-private for a region). In the same way, the IPD-IMGT/HLA Allele Ethnicity Tool (49) was used to retrieve information on the reported ethnicity of HLA subtypes.

### Univariable and multivariable analyses of HLA-HIV clinical parameter associations

HLA associations with the two HIV clinical parameters defined to be independently predictive of HIV disease progression were investigated. These included HIV pVL (50) and absolute CD4 count (51), used in routine clinical monitoring of infection in people living with HIV. Concisely, HLA subtypes with an observed frequency equal to or greater than 3 were selected to evaluate HLA-HIV associations; these included 99 HLA subtypes (27 *HLA-A*, 47 *HLA-B*, and 25 *HLA-C*). Univariable linear regression analyses were used to evaluate associations between each HLA subtype (treated as a binary variable, i.e., comparing A*01:01^+^ vs A*01:01 ^−^ individuals) and the parameter of interest. A multivariable linear regression model was constructed to account for potential confounders of HLA-HIV associations. Briefly, independent models were constructed relating HLA subtypes to HIV pVL or CD4 counts while adjusting for age, gender, recruitment city (coded as *n*−1 binary variables), HIV incidence (recent vs established infection, coded as a binary variable), CCR5 genotype, and the effect of the significant HLA-HIV associations for that parameter (defined as the HLA subtypes with *P* < 0.05 in the corresponding linear regression univariate analysis). The selection of “optimal” models was carried out with the aid of the stepwise algorithm [stepAIC function from MASS package (52) and train function from caret package (53)] in R. Specific covariates and HLA subtypes included in each model are listed in the corresponding Online Table Legend. Multiple comparisons were addressed with the false discovery rate (*q*-values, using the R package FDRestimation) (54, 55), where associations with *P* < 0.05 and *q* <0.2 were considered to be significant. Statistical analyses were undertaken using R v4.2.1 (56).

## RESULTS

### Honduras HIV cohort clinical and demographic characteristics

**FIG 1:**
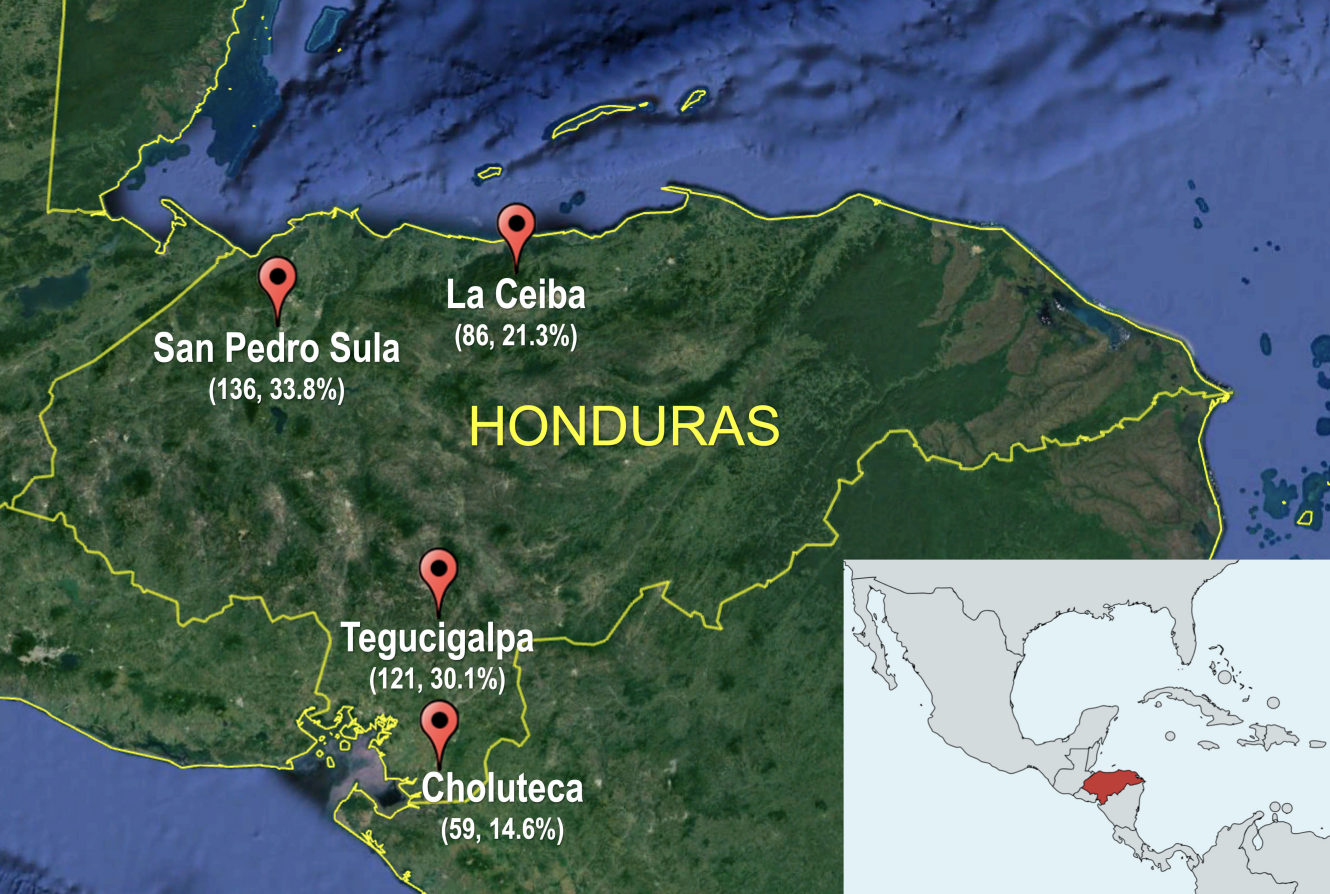
Geographical residence of the individuals recruited in the study. The map shows the four Honduras cities where the participants were recruited, including Tegucigalpa (14°6′N, 87°13′W), San Pedro Sula (15°30′N, 88°2′W), La Ceiba (15°46′N, 86°50′W), and Choluteca (13°18′N, 87°11′W). The number and percentage (*N*, %) of participants from each city are denoted. The map was generated using Google Earth Pro for macOS (https://www.google.com/intl/es-419_ALL/earth/versions/#earth-pro).

The cross-sectional Honduras HIV cohort comprised 402 HIV-1 B clade-infected, ART-naïve individuals recruited in four Honduran cities, including Tegucigalpa (*n* = 121, 30.1%), Choluteca (*n* = 59, 14.6%), San Pedro Sula (*n* = 136, 33.8%), and La Ceiba (*n* = 86, 21.3%; Fig. 1). As previously observed in Latin American HIV cohorts, the clinical and demographic characteristics of the Honduras cohort are coherent with the frequent diagnosis of HIV in the advanced stage of infection (57) (Table 1). Overall, the participants’ median age at enrolment was 34 years old [interquartile range (IQR) 26–42]; a little more than half were male (56.9%); median HIV pVL was 4.46 log^10^ RNA copies/mL (IQR 3.6–5.1); and median CD4 count was 297.5 cells/µL (IQR 96–509). The most common risk factors for HIV acquisition were being heterosexual (78%) and MSM (14%); most of the participants were single (57%) or had a domestic partner (30%); and almost half of them were unemployed (49%). Regarding education, 77% of participants attended elementary (48%) or secondary school (28%), and 10.4% of individuals were not proficient in reading or writing.

**TABLE 1:** Demographic and clinical characteristics of HIV-infected Honduras individuals^*a*^

	Honduras HIV cohort
*N*	402
Age [years, median (IQR)]	34 (26–42)
Female [*N* (%)]	173 (43.03)
Log^10^ HIV plasma viral load [RNA copies/mL, median (IQR)]	4.46 (3.61–5.16)
CD4^+^ T cell count [cells/μL, median (IQR)]	297.5 (96.0–509.2)
HIV risk factor [*N* (%)]	
Heterosexual	315 (78.3)
Men who have sex with men	57 (14.1)
Bisexual	13 (3.2)
Injecting drug users	6 (1.4)
Mother-to-child transmission	1 (0.2)
Unknown	10 (2.4)
Marital status [*N* (%)]	
Single	232 (57.7)
Domestic partner	123 (30.5)
Married	38 (9.4)
Unkown	9 (2.2)
Employment [*N* (%)]	
Unemployed	200 (49.7)
Employed	165 (41.0)
Student	20 (4.9)
Unknown	17 [4.2]
Education [*N* (%)]	
Elementary school	196 (48.7)
Secondary school	114 (28.3)
Degree or technical qualification	46 (11.4)
Illiterate	42 (10.4)
Unknown	4 (0.9)
Recruitment city [*N* (%)]	
San Pedro Sula	136 (33.8)
Tegucigalpa	121 (30.0)
La Ceiba	86 (21.3)
Choluteca	59 (14.6)

^*a*^ IQR, interquartile range.

### CCR5-Δ32 frequency and its impact on HIV clinical parameters

**FIG 2:**
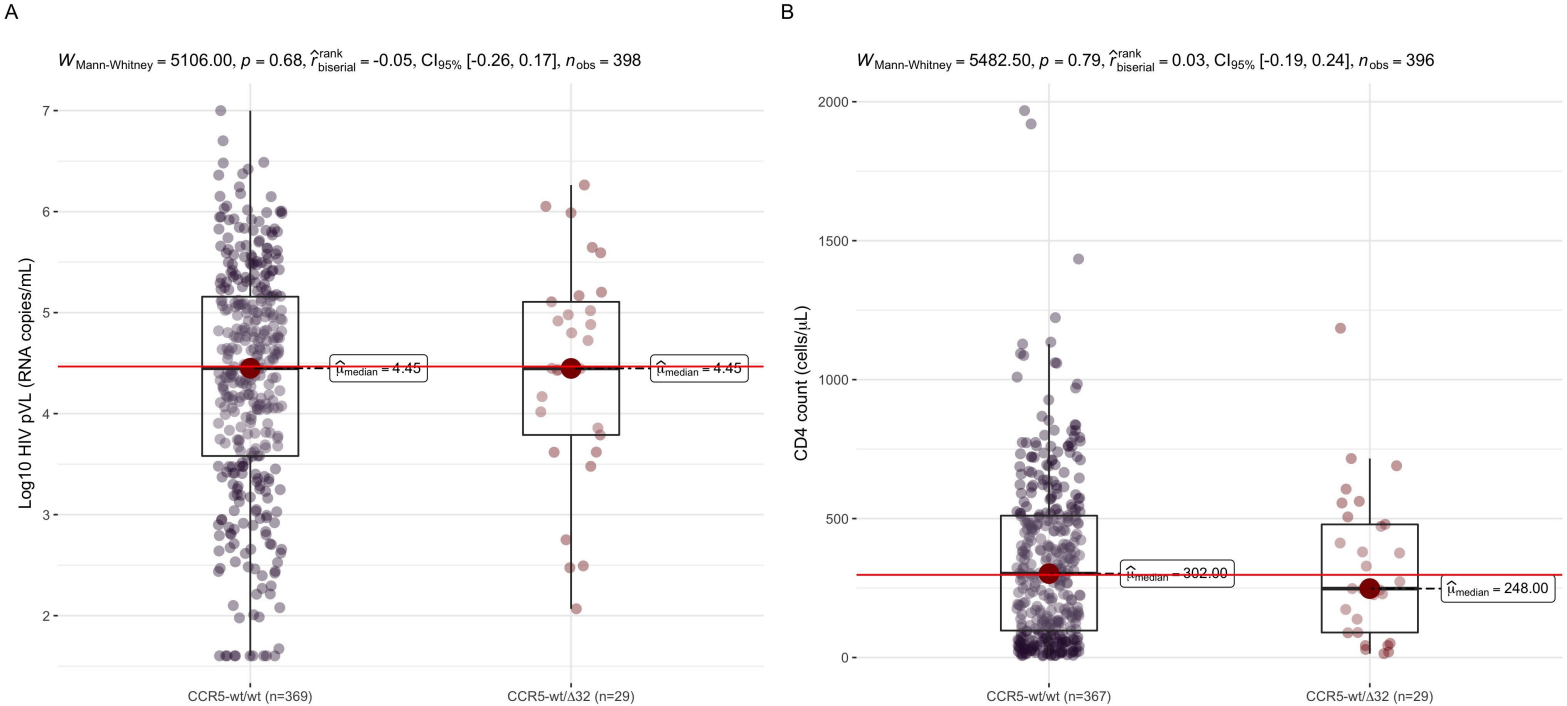
HIV clinical parameter comparisons between CCR5 genotypes. The plot shows HIV pVL (panel A) and CD4 T cell count (panel B) comparisons between CCR5-wt/wt and CCR5-wt/Δ32 genotypes. Differences were evaluated using the two-tailed Mann-Whitney *U* test. The horizontal red lines indicate the overall HIV pVL and CD4 count medians; horizontal lines within boxes indicate the median (Q2); boxes comprise the Q1 and Q3 quartiles (IQR), while the extreme lines (whiskers) show Q3 + 1.5 × IQR to Q1 − 1.5 × IQR of values. Figures and statistics were done in R using the ggstatplot package (58).

The AF (denominator 2*n*) of the CCR5-Δ32 deletion in the Honduras cohort was scarce (AF = 0.036), while the non-truncated variant CCR5-wt (wild type) was the most common (AF = 0.964). Concisely, 29 (out of 402) individuals were heterozygous for the CCR5-wt/Δ32 genotype [genotype frequency (GF) = 0.072; Table 2], whereas 373 individuals were positive for the CCR5-wt/wt genotype (GF = 0.927; Table 2). No homozygous individuals were found for the CCR5-Δ32 mutation. We did not witness a significant difference in GF between the recruitment cities (Fisher’s exact test, *P* = 0.6313; Table 2). No impact on HIV pVL and CD4 counts was distinguishable between the individuals with the CCR5-wt/wt and those with the CCR5-wt/Δ32 genotype [non-parametric two-tailed Mann-Whitey *U* test: *P* = 0.68 and 0.79 for pVL and CD4, respectively, see Fig. 2; univariable linear regression: *P* = 0.66 and 0.70 for pVL and CD4, respectively (data not shown)].

**TABLE 2:** CCR5 genotype frequency in HIV-infected Honduran individuals

GF (*N*)^*b*^	Whole Honduras	Choluteca	La Ceiba	San Pedro Sula	Tegucigalpa	*P*-value^*a*^
**CCR5-wt/wt**	0.927 (369)	0.949 (56)	0.928 (78)	0.902 (121)	0.942 (114)	0.6313
**CCR5-wt/Δ32**	0.072 (29)	0.050 (3)	0.071 (6)	0.097 (13)	0.057 (7)	

^*a*^ *P*-value calculated comparing CCR5 genotype counts between recruitment Honduran cities using Fisher’s exact test. None of the participants were homozygous for the CCR5-Δ32 mutation.

^*b*^ Genotype frequency (GF) of CCR5 variants were calculated as direct count (denominator n).

### HLA class I allele diversity

**FIG 3:**
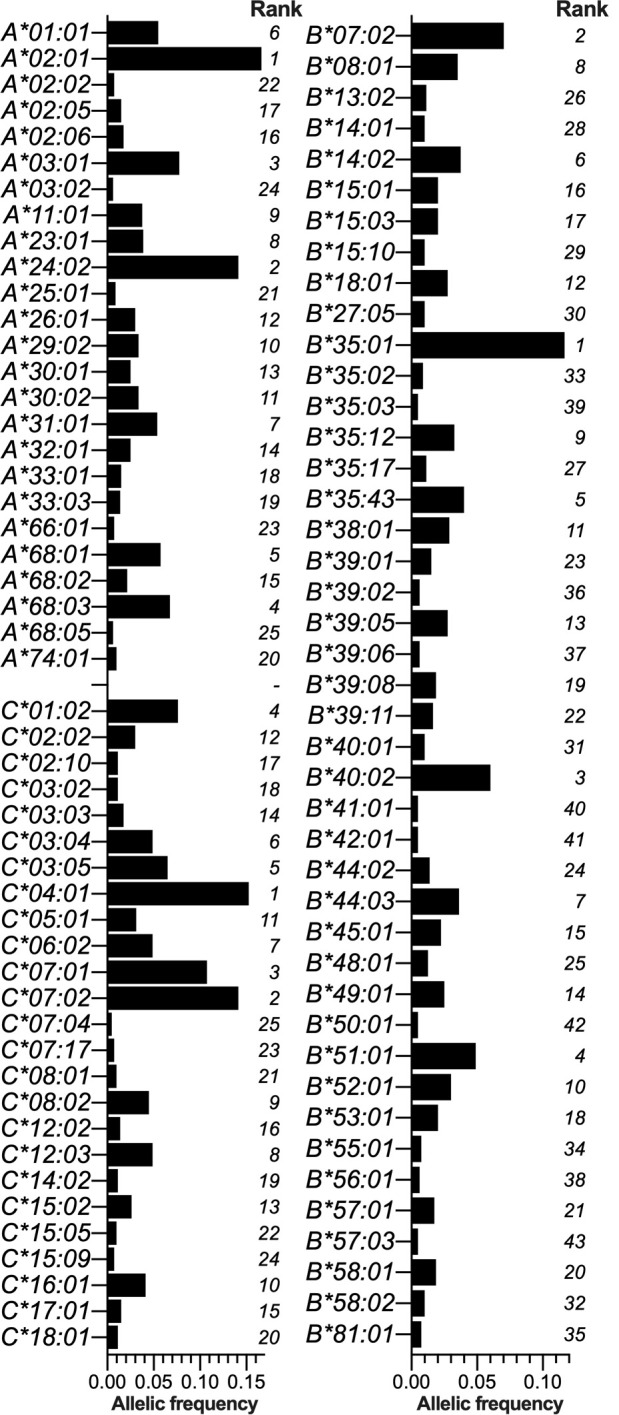
HLA class I (*HLA-A*, *HLA-B*, and *HLA-C*) AFs in the Honduras HIV cohort. AFs were calculated by direct count (denominator 2*n*) in the Mestizo Honduras cohort (*n* = 402). Only HLA subtypes with AF equal to or greater than 0.005 (*n* ≥ 4) are shown (including 25 *HLA-A*, 43 *HLA-B*, and 25 *HLA-C*). The frequency rank of each HLA subtype in each locus is denoted.

HLA cI allele expression was assessed using 402 non-related individuals from the Honduras HIV cohort genotyped by Sanger sequencing to assign second-field resolution HLA cI subtypes. A total of 140 distinct subtypes across loci were identified in the present cohort, including 42 *HLA-A*, 29 *HLA-C*, and 69 *HLA-B* distinct subtypes (Table S1), whose frequency has not been reported in any Honduras population so far. Genotype frequencies of the HLA cI loci marginally deviate from Hardy-Weinberg expectations (Table S2). Allele frequencies of *HLA-A*, *HLA-B*, and *HLA-C* are listed in Fig. 3; Table S3, in which the following subtypes were the most frequent: A*02:01 (AF = 0.167), A*24:02 (0.140), A*03:01 (0.077), A*68:03 (0.067), A*68:01 (0.057), A*01:01 (0.054), and A*31:01 (0.053); B*35:01 (0.118), B*07:02 (0.069), B*40:02 (0.059), B*51:01 (0.048), B*35:43 (0.039), B*14:02 (0.037), and B*44:03 (0.036); and C*04:01 (0.151), C*07:02 (0.141), C*07:01 (0.107), C*01:02 (0.075), C*03:05 (0.064), C*03:04 (0.049), and C*06:02 (0.049), respectively.

Next, we aimed to investigate if distinct HLA expression patterns exist between enrolment Honduran cities; to accomplish the latter, we performed a pairwise comparison of AF between those alleles with AF >0.005 in at least one city ( Fig. S1). In summary, 17 subtypes (i.e., 15.1% of a total of 112 subtypes, including 6 *HLA-A*, 5 *HLA-C*, and 6 *HLA-B*) showed significant AF differences between at least two cities. Overall, the following HLA subtypes were observed in significantly higher frequency in the following recruitment cities: Tegucigalpa: A*01:01, C*08:02, B*14:02, and B*35:43 (Amerindian); San Pedro Sula: A*33:03, B*39:08 (Amerindian), and B*57:01 (Caucasian); La Ceiba: A*30:01, A*68:02, C*04:01, C*17:01, and B*53:01 (African); and Choluteca: A*30:02, A*68:05, C*01:02, C*03:05, and B*40:02 (Amerindian).

### Distinctive immunogenetic profile of HIV-infected individuals from Honduras: HLA haplotype diversity

**FIG 4:**
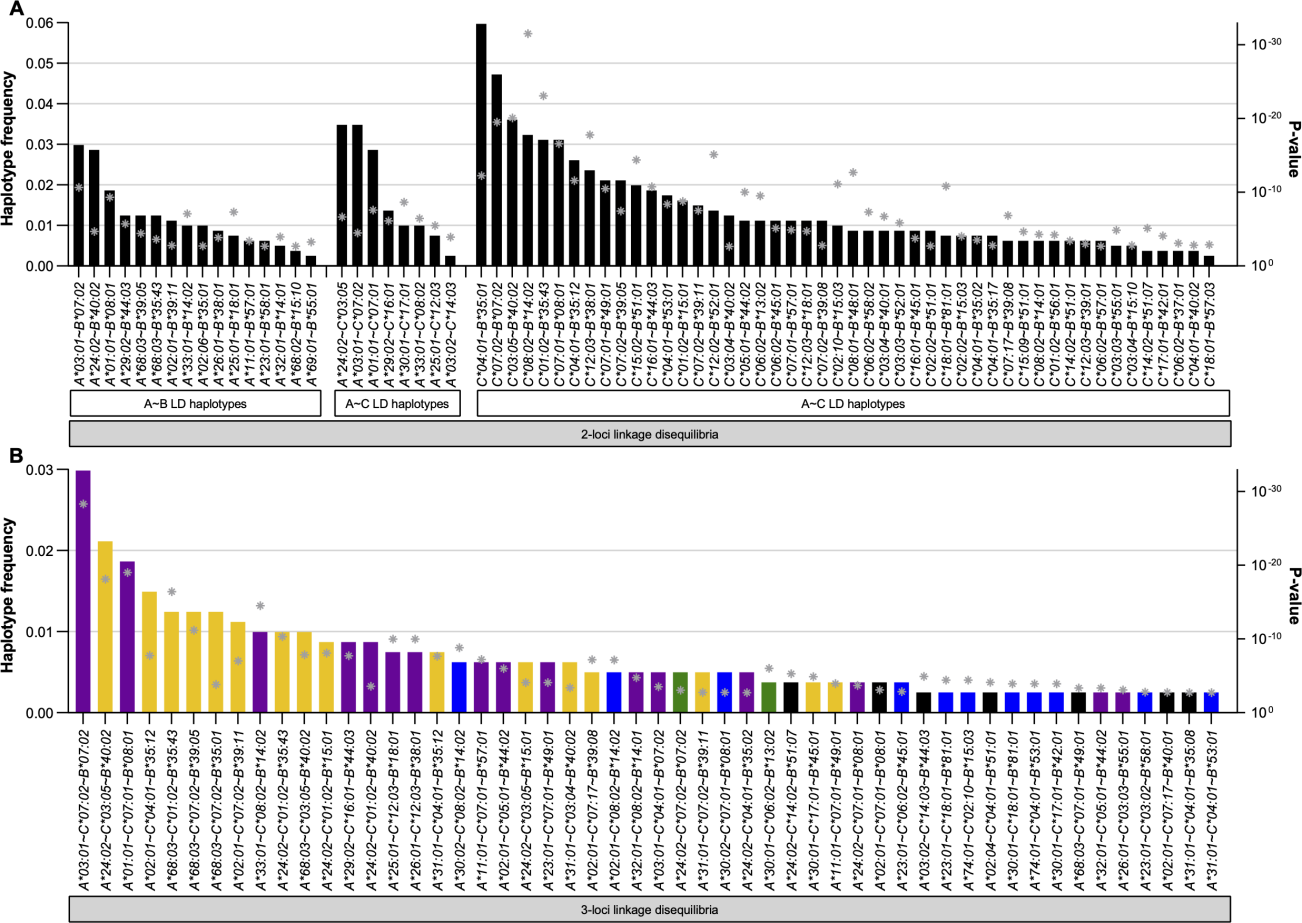
Frequent two- and three-loci HLA cI haplotype structures in the Honduras HIV cohort. Two-loci (panel A) and three-loci (panel B) HLA haplotype structures are shown. LD between HLA allele pairs was assessed using Fisher’s exact tests with multiple comparisons addressed via false discovery rate (*q*-value). For two-loci LD analysis, 6,117 distinct two-way tests were performed (*P* < 0.05 and *q* <0.2 were considered significant). Haplotype frequencies were calculated by direct gene count (denominator 2*n*; left y axis). Overall, 72 two-loci haplotype structures are shown (panel A), including 16 for *A*–*B*, 8 for *A*–*C*, and 48 for *B*–*C*. For each of the statistically significant HLA pairs discovered in the two-loci LD analysis, a Fisher’s test is performed against the third loci (3,032 distinct two-way tests, *P* < 0.05 and *q* <0.2 were considered significant), encompassing 52 three-loci (*A*–*C*–*B*) haplotypes (panel B). MPA (i.e., putative origin; see Materials and Methods) was determined for every three-loci haplotype structure: Caucasian MPA (purple), Amerindian MPA (yellow), African MPA (blue), Oriental MPA (green), and not determined or mixed origin (black). *P*-values of Fisher’s exact tests [right y axis (values are reversed, so higher gray asterisks are more significant)] of HLA haplotypes are denoted.

**FIG 5:**
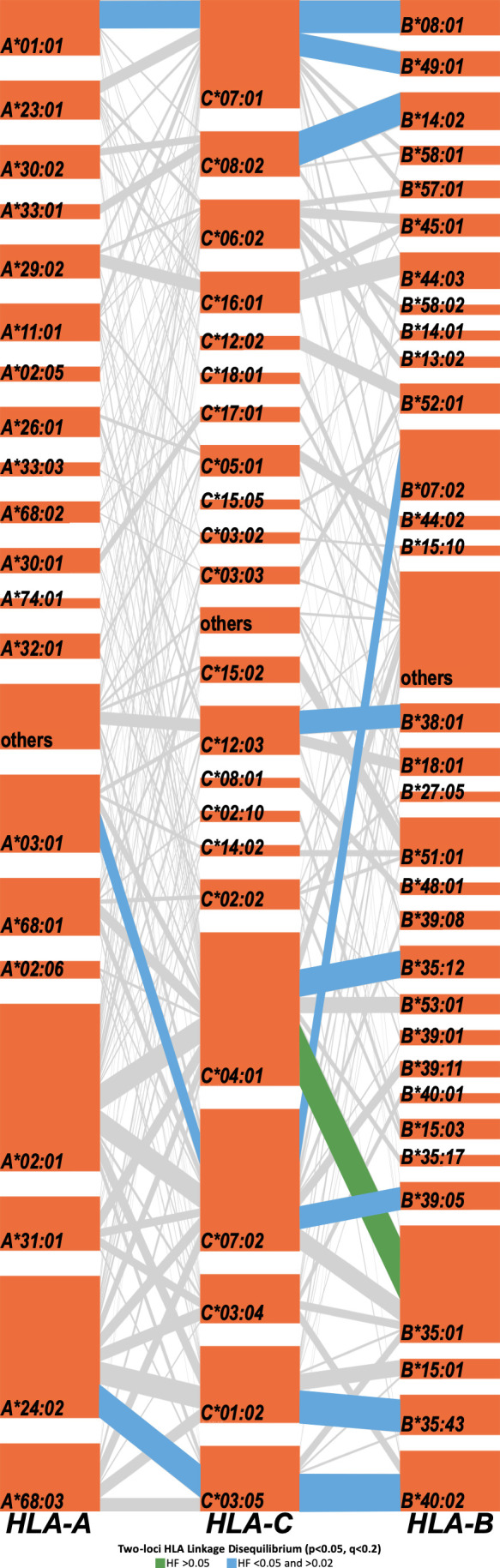
HLA class I haplotype structures and LDs in the Honduras HIV cohort. The high-dimensional visualization tool Disentangler was used to graph HLA haplotype structures in a textile plot. HLA loci are stacked vertically, with each orange tile representing a specific HLA subtype and with segments connecting linked subtypes on adjacent loci. The height of each tile and the thickness of each segment correspond to HLA allele and haplotype frequencies, respectively. The most frequent HLA allele pairs (two-loci) found to be in LD are denoted in green (HF >0.05) and blue (HF <0.05 and >0.02), and less frequent pairs (HF <0.02) are shown in gray. Frequently linked (HF >0.02) HLA-A–B allele pairs were also found in the cohort, including A*03:01–B*07:02 (HF = 0.029) and A*24:02–B*40:02 (HF = 0.028), not shown in the figure (see Online Supplementary Table S4).

Strong linkage disequilibria among HLA cI loci allowed us to distinguish frequent two- and three-loci haplotype structures in the Honduras cohort (Fig. 4; Tables S4 and S5). A total of 72 distinct two-loci HLA haplotype structures were identified (*P* < 0.05 and *q* <0.2; Fig. 4A; Table S4), including 16 for *HLA-A–B*, 8 for *HLA-A–C*, and 48 for *HLA-C–B*, of which only 1 pair had haplotype frequency (HF) >0.05 [C*04:01–B*35:01 (HF = 0.059), see green segment in Fig. 5); 14 haplotypes had HF <0.05 and >0.02 (A*03:01–B*07:02, A*24:02–B*40:02, A*24:02–C*03:05, A*03:01–C*07:02, A*01:01–C*07:01, C*07:02–B*07:02, C*03:05–B*40:02, C*08:02–B*14:02, C*01:02–B*35:43, C*07:01–B*08:01, C*04:01–B*35:12, C*12:03–B*38:01, C*07:01–B*49:01, and C*07:02–B*39:05; see blue segments in Fig. 5).

Likewise, 52 distinct three-loci HLA haplotype structures were identified in the present cohort (Fig. 4B and Table S5, *P* < 0.05 and *q* <0.2), in which haplotypes with Amerindian MPA [i.e., as those reported in reference (48) in high relative frequency in Amerindian populations; see Materials and Methods] featured prominently [e.g., 16 haplotypes (30.7% of 52): A*24:02–C*03:05–B*40:02, A*02:01–C*04:01–B*35:12, A*68:03–C*01:02–B*35:43, A*68:03–C*07:02–B*39:05, A*68:03–C*07:02–B*35:01, A*02:01–C*07:02–B*39:11, A*24:02–C*01:02–B*35:43, A*31:01–C*04:01–B*35:12, A*02:01–C*07:17–B*39:08, A*31:01–C*07:02–B*39:11, among others, see yellow bars in Fig. 4B]. As expected for Mestizos in Central America, a group of HLA haplotype structures with Caucasian/European MPA were found [e.g., 16 haplotypes (30.7% of 52): A*03:01–C*07:02–B*07:02 (cohort most frequent haplotype), A*01:01–C*07:01–B*08:01, A*33:01–C*08:02–B*14:02, A*29:02–C*15:01–B*44:03, among others, see purple bars in Fig. 4B], and in lesser extent, haplotype structures described in Oriental populations [2 haplotypes (3.84% of 52); green bars in Fig. 4B). Remarkably, an important proportion of HLA haplotypes with African MPA were observed in the Honduras cohort [e.g., 11 haplotypes (21.1% of 52): A*25:01–C*12:03–B*38:01, A*30:02–C*08:02–B*14:02, A*02:01–C*08:02–B*14:02, A*30:02–C*07:01–B*08:01, A*23:01–C*05:02–B*45:01, among others, see blue bars in Fig. 4B), being congruent with the description of Garifunas and Afro-descendants individuals in the country.

To further validate the three-loci HLA haplotype structures, we estimate HLA HF using the EM algorithm in Arlequin v3.5 (45). HFs derived from EM analysis were highly concordant with those obtained by Fisher’s exact tests (Spearman correlation, rho = 0.9095, *P* < 0.0001; Fig. S2). Overall, these findings emphasize the HLA immunogenetic uniqueness of the Honduras HIV cohort, which not only expresses Amerindian and Caucasian alleles and haplotypes but also distinguishes a high proportion of African-descendant subtypes, and support it as ideal for identifying novel HLA correlates of HIV control.

### HLA associations with HIV pVL and CD4 counts in the Honduras HIV cohort

**FIG 6:**
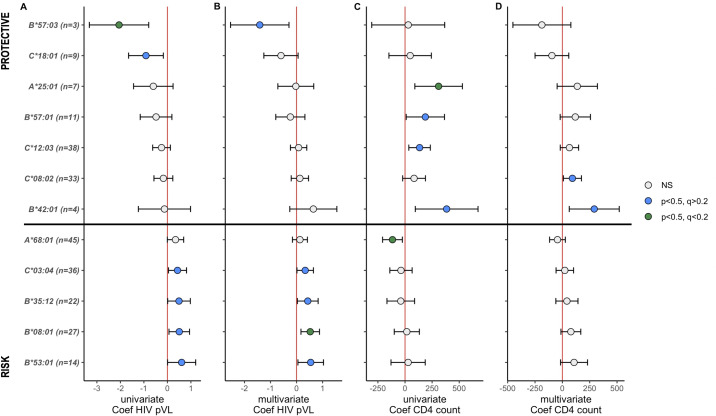
Summary of univariable and multivariable analyses of HLA-HIV associations using HIV pVL (**A and B**) and CD4 counts (**C and D**) in the Honduras cohort. Only HLA subtypes with at least one significant association (*P* < 0.05) in univariable (**A and C**) or multivariable (**B and D**) linear regression analyses are shown (see Materials and Methods). Linear regression coefficients and 95% confidence intervals are shown. HLA subtypes are characterized by univariable pVL coefficients, in which protective HLA-HIV associations are shown at the top and detrimental/risk associations at the bottom. The number (*n*) of individuals expressing each HLA subtype is shown. Red vertical lines denote a coefficient equal to zero. A complete list of univariate and multivariate HLA-HIV associations is denoted in Table S6.

Agreeing with the unique HLA class I expression in the Honduras HIV cohort presented above and that distinct HLA distribution can drive distinct HIV control patterns at the population level, we aimed to assess HLA expression in relation to HIV clinical parameters canonically and independently associated with HIV disease outcome. We explored the latter by quantifying the effects of HLA subtypes on both HIV pVL and CD4 counts in a univariable linear regression analysis. We next achieved a multivariable analysis to adjust for cofounding variables (age, gender, recruitment city, HIV incidence, CCR5 genotype, and the effect of significant HLA-HIV associations for each parameter; see Materials and Methods). Subsequent linear regression coefficients and 95% confidence intervals of HLA subtypes with at least one significant association (univariable or multivariable) with HIV pVL or CD4 counts are summarized in Fig. 6, and the whole catalog of linear regression statistics of all HLA subtypes (*n* ≥ 3) are exhibited in Table S6.

Overall, HLA-HIV significant associations in univariable and multivariable were concordant, though some exceptions were distinguished (particularly in the CD4 analysis, Fig. 6). At the stated threshold of *P* < 0.05, we observed six HLA subtypes (four *HLA-B* and two *HLA-C*) associated with HIV pVL distinct outcomes and six subtypes (two *HLA-A*, two *HLA-B*, and two *HLA-C*) associated with CD4 counts.

Being consistent with earlier studies in Afro-American and African HIV cohorts, the canonically HLA-HIV associations linked to the protective B*57:03 (which differs by two residues from Caucasian B*57:01) (7, 9, 17 – 19, 59) and the detrimental B*53:01 (related to the B*35 risk allele group) (10, 60) were found in this HIV cohort, being associated with the lowest and highest HIV pVL, respectively (coefficients: −2.0 and 0.66, respectively; in all cases *P* < 0.05; Fig. 6A). We also perceived that the previous C*18:01 protective association (15, 18, 61, 62), a subtype that has been described in LD here (Fig. 4A) and elsewhere with both African B*57:03 and B*81:01 (also portrayed as protective) (7, 13, 18, 62), was related to lower HIV pVL (coefficient: −0.91; *P*-value: 0.015).

Regarding the CD4 count analyses, previous HLA-HIV associations were distinguished, encompassing the earlier described protective A*25:01, B*57:01, C*12:03, and B*42:01 subtypes (3, 7, 10, 12, 13, 17, 18, 63 – 66) and the detrimental A*68:01 subtype (18, 62), being associated with higher and lower CD4 counts, respectively (Fig. 6C and D). Markedly, the C*08:02 subtype (in strong LD with B*14:01/02, see Discussion) was associated in multivariable analysis with higher values of CD4 counts (coefficient: 91.8; *P*-value: 0.029).

Noteworthy to mention, the Amerindian B*35:12 subtype [common *HLA-B* in the cohort (AF = 0.032, rank ninth), rarely or not observed in non-Mestizo populations from Mesoamerica) was associated with higher HIV pVL values in both linear regression univariable and multivariable analyses (0.49 and 0.43 log^10^ pVL increase, respectively, with respect to B*35:12 negative individuals; in all cases, *P* < 0.05) and with lower CD4 counts [univariable coefficient: −39.2, *P* = 0.054 (did not reach statistical significance)]. Other HLA-HIV associations not previously described elsewhere were C*03:04 and B*08:01, which in both univariable and multivariable analyses were associated with higher HIV pVL (coefficients: 0.43 and 0.33, respectively; in all cases, *P* < 0.05; Fig. 6; Table S6).

## DISCUSSION

In the present study, managed among the cross-sectional Honduras HIV cohort (*n* = 402), to our knowledge, this is the first comprehensive description of HLA class I loci and CCR5 expression in Honduras. We have described frequencies of HLA subtype and haplotype structures, CCR5-Δ32 and CCR5-wt allelic and genotype distribution, and defined both previously reported and new associations between HLA subtype expression and the HIV clinical parameters correlated to HIV disease outcome (i.e., HIV pVL and CD4 counts).

As expected for a non-Caucasian cohort (28 – 32), the allele frequency of the CCR5-Δ32 deletion (related to a better HIV outcome) was low (AF = 0.036), while the non-truncated CCR5-wt variant was the most common (AF = 0.964). Regarding genotype frequency (denominator *n*), a minor proportion of individuals were heterozygous for the CCR5-wt/Δ32 genotype (GF = 0.072), whereas the majority were homozygous for the non-truncated variant (CCR5-wt/wt, GF = 0.927; Table 2). No homozygous subjects were observed for the 32-bp deletion (CCR5-Δ32/Δ32 genotype), which is not surprising considering the overall low frequency of this deletion in the present study. When we weighed the HIV clinical parameters related to HIV disease outcome (i.e., HIV pVL and CD4) between the subjects carrying the CCR5-wt/Δ32 and CCR5-wt/wt genotypes, we did not observe any significant difference (Fig. 2). The CCR5-Δ32 impact over clinical parameters was also evaluated using CCR5-Δ32 as a binary covariate in the subsequent HLA-HIV multivariable analysis. Nevertheless, as the CCR5-Δ32 covariate did not contribute to improving both pVL and CD4 linear regression model accuracies (i.e., it did not show the effect of the model’s adjusted *R* squared and the covariate *P*-value was never <0.1, concluding that the covariate did not contribute to explaining the variance of the dependent variable), consequently, the CCR5-Δ32 covariate was removed from both final multivariable models. Previous inconsistencies in HIV control between the occurrence of CCR5-Δ32 genotypes have been observed in distinct cohorts studied, probably due to the presence or emergence of CXCR4-tropic HIV strains, commonly observed in chronic HIV cohorts like the present here (median CD4 = 297.5 cells/µL) or due to the CCR5-Δ32 scarce frequency in the studied population (only 29 individuals were heterozygous for the CCR5-wt/Δ32 genotype and none was positive for the CCR5-Δ32/Δ32 genotype; so we cannot rule out insufficient statistical power to detect its effect).

As previously portrayed for Mestizo individuals in the Mesoamerica region, we observed an expansion of HLA subtypes that are prevalent in Mesoamerican Amerindians, being endemic in the region or rarely observed elsewhere (21), including HLA-A*02:06, A*31:01, A*68:01/03/05, B*35:12/17/20/43, B*39:02/05/06/08, B*52:01, C*03:05, and C*15:09 (Fig. 3). When we explored if a regional HLA distribution pattern exists between the Honduran recruitment cities, we perceived a substantial increase of the Amerindian/Hispanic B*35:43 subtype in Tegucigalpa and B*39:08 in San Pedro Sula (both subtypes are endemic in Mesoamerica, in particular in Central America countries) (20, 21, 48); we also observed a significant increase of the African B*53:01 in Choluteca and the Caucasian B*14:02 and B*57:01 subtypes in Tegucigalpa and San Pedro Sula, respectively ( Fig. S6). These outcomes denote the subtle differences that exist in HLA frequency distribution in the four recruitment Honduras cities and reflect the contribution of Amerindian, African, and Caucasian subtypes to the genetic HLA class I makeup of the Honduras Mestizo population.

Given the strong linkage disequilibria that exist between HLA cI loci and despite the relative low number of unrelated individuals, we were able to statistically identify both two- and three-loci HLA haplotype structures. Overall, 72 distinct two-loci haplotype structures were found, and being congruent with the closer proximity (~100 kb) among *HLA-C* and *HLA-B* loci within the MHC beta gene cluster, 48 (66%) haplotypes were found for *HLA-C–B* [being the most frequent C*04:01–B*35:01 (HF = 0.059)]; in contrast, only 16 (22%) were found for *A*–*B* haplotypes and 8 (11%) for *A*–*C* haplotypes (Fig. 4 and 5; Table S4). Similarly, 52 distinct three-loci HLA haplotype structures were detected using Fisher’s exact tests (see Materials and Methods), and these were highly concordant with those obtained with the Arlequin’s EM algorithm (Spearman correlation, rho = 0.9095, *P* < 0.0001; Fig. S2), validating that both methods can be used indistinctively to investigate three-loci HLA cI haplotypes in our study cohort.

To our knowledge, this is the first report in a Honduras population assessing ancestry estimation using HLA haplotype structures. Notably, when assessing the MPA (i.e., putative origin) of the latter three-loci haplotype, we found that 30.7% (16 haplotypes) were of Amerindian MPA, 30.7% (16 haplotypes) of Caucasian MPA, 21.1% (11 haplotypes) of African MPA, and, to a lesser extent, 3.8% (2 haplotypes) of Oriental MPA. Among the 16 Amerindian MPA haplotypes found here, the following were observed in earlier studies in Amerindians or in admixed Latin America Mestizo cohorts [including those reported in the United States of America (USA) Hispanic population]: A*24:02–C*03:05–B*40:02 described in Costa Rica Amerindians and Nicaragua Mestizos (67) as part of an extended haplotype with DRB1*04:07, and in Hispanic population in the USA National Marrow Donor Program (NMDP) (68); A*68:03–C*01:02–B*35:43 in Nicaragua Mestizos (67); A*68:03–C*07:02–B*39:05 and A*68:03–C*07:02–B*35:01 in Mayans from Chiapas (Mexico) (69); A*02:01–C*07:02–B*39:11 in Mestizos from Costa Rica (67) and Colombia (Bogota Cord Blood Bank) (70); A*24:02–C*01:02–B*35:43 in Amerindian North Wiwa El Encanto (Colombia) (71) and Costa Rica Amerindians (67); A*68:03–C*03:05–B*40:02 in Mestizos from Managua (Nicaragua) (72); A*24:02–C*01:02–B*15:01 in Tarahumaras (Amerindians from Mexico) (73) and in Amerindian North Wiwa El Encanto (Colombia) (71); A*31:01–C*04:01–B*35:12 in Caribbean Indian (North America Amerindian, USA NMDP) (74) and in Mexico City Mestizos [reported in reference (48)]; A*24:02–C*03:05–B*15:01 in Mestizos from Managua (Nicaragua) (72); A*31:01–C*03:04–B*40:02 in Lacandon Mayans (Chiapas, Mexico) (69) and in Mexico City Mestizos (75); A*02:01–B*39:08–C*07:17 in USA Hispanic population (76); A*31:01–C*07:02–B*39:11 in Mestizos from Managua (Nicaragua) (72) and in USA Hispanic population (76); A*30:01–C*17:01–B*45:01 in admixed population from Brazil (Vale do Ribera Quilombos) (77) and in Hispanic population from Panama (78); among others.

Remarkably, an important proportion of African MPA haplotypes were found, encompassing 11 out of 52 (21.1%) three-loci haplotypes previously described in high relative frequency in the following Black populations: A*30:02–C*08:02–B*14:02 in Brazil [Brazil Rio de Janeiro Black and Parda populations in reference (48)], in Luo Tribe in Kenya (Nyanza Province) (79), and in South Africa (80); A*02:01–C*08:02–B*14:02 in Brazil [Barra Mansa Rio State Black in reference (48)] and in Uganda Kampala population (81); A*23:01–C*06:02–B*45:01 in South Africa (82) and in Luo Tribe (Kenya) (79); A*23:01–C*18:01–B*81:01 in Costa Rica African-Caribbeans (67) and in Tanzania Massai population [defined in reference (48)]; A*74:01–C*02:10–B*15:03 in Luo Tribe in Kenia (79) and in Amerindians from Colombia (North Chimila) (83); A*30:01–C*18:01–B*81:01 in Luo Tribe in Kenia (79) and in USA African Americans (84); A*74:01–C*04:01–B*53:01 in admixed population from Brazil (Vale do Ribera Quilombos) (77) and in Rio de Janeiro Parda (Brazil) [described in reference (48)]; among others.

In spite of the cross-sectional approach, the HIV disease chronicity, and the relatively small number of individuals investigated, we have detected previously reported HLA-HIV associations in Caucasian, African-American, and African cohorts, comprising the protective associations of B*57:03 (associated with HIV pVL) and A*25:01, B*57:01, C*12:03, and B*42:01 (related to CD4 counts) and the risk associations with B*53:01 (HIV pVL) and A*68:01 (CD4 count), accomplishing the accuracy of our univariable and multivariable analyses and increasing the certainty of the new HLA-HIV found in the Honduras HIV cohort. We have confirmed the detrimental association of the Amerindian B*35:12 subtype in both univariable and multivariable analyses. This common risk subtype was identified in previous research by our research group in a large cohort of Mexico and Central America (*n* = 3,213) (20) and portrayed as a putative new member of the B*35 Px HIV risk subtype group (85). Validation of this Amerindian subtype, consistently associated with both higher HIV pVL and lower CD4 counts, warrants the elucidation of the possible immune mechanisms associated with the disadvantageous HIV outcome of Latin American B*35:12-positive individuals. Other HLA-HIV associations not previously observed elsewhere were C*03:04 (LD with Amerindian A*31:01–B*40:02) and B*08:01 (LD with A*02:01–C*07:01, the second most frequent Caucasian MPA haplotype), which in both univariable and multivariable analyses were associated with higher HIV pVL (Fig. 6).

There are a number of limitations and caveats worth mentioning. Due to the relatively small number of individuals in each of the four recruitment cities, we were not confident enough to assess HLA haplotype structures in individual enrollment places. Thus, the evaluation of HLA haplotype MPA was not possible between cities. Only minor differences in HLA distribution could be observed when we made a comparison of HLA allele frequencies (see above). We are aware that bias in HLA distribution might exist, given that we scrutinized individuals living with HIV. Nevertheless, the entity of HLA haplotype structures remains intact.

Regarding HLA-pVL/CD4 associations, given the roughly complete LD between particular HLA subtypes, we were unable to disentangle HLA-HIV associations between HLA subtypes previously reported to be associated with HIV outcome. In particular, the C*08:02 subtype was associated in multivariable analysis with CD4 count higher values (coefficient: 91.8; *P*-value: 0.029). This *HLA-C* subtype is in strong LD with B*14:02 [LD *P*-value = 2.79E−32, the most significant two-loci LD pair in this cohort (i.e., roughly all B*14:02 subjects were also C*08:02 positive), see Fig. 4A] and B*14:01 (LD *P*-value = 5.17E−05), both alleles previously described as protective (17). Nonetheless, these *HLA-B* subtypes do not reach statistical significance in our analyses (may be due to the lack of statistical power due to the low number for individual *HLA-B* subtype). Another example is C*18:01 (associated with lower pVL) that is in strong LD with B*57:03 (also associated with pVL) and B*81:01 (not a significant association but previously associated with protection). Furthermore, we cannot exclude the possibility of previously described additive or synergistic effects between HLA alleles (18). Unfortunately, we cannot test this hypothesis with such extensive LD between subtypes and a low number of positives for every HLA subtype.

With such a modest sample size, we were able to find statistically significant associations and confirm previously reported associations with HLA class I alleles in other populations and confirmed Amerindian HLA-HIV associations previously described in larger cohorts. Nonetheless, not all significant HLA-HIV associations reported here survive multiple comparison correction (*q* < 0.2; see blue dots in Fig. 6) in our analyses, meaning that insufficient statistical power was encountered given such a large set of comparisons (i.e., a large number of HLA subtypes with enough observations to be tested in the Honduras cohort). Finally, the possibility of spurious associations is always a concern in HLA-HIV association investigations, particularly when reporting new associations in HLA landscapes that show endemic subtypes like the present here (e.g., Amerindian C*03:04 associated with higher pVL). Hence, validation of these new associations is needed in extended cohorts in Honduras or in other Latin American populations that exhibited similar HLA subtype distributions, along with elucidation of the possible immune mechanisms as warranted. In spite of these caveats, the present study confirms that some HLA-HIV associations (e.g., B*57:01, B*57:03, and B*53:01) excel at the boundaries of genetic admixture of the populations studied, while others are likely to be exclusive (e.g., Amerindian B*35:12 subtype) given the exceptional HLA immunogenetic background of the Honduras population.

An important contribution of this study was to provide data on second-field (i.e., four-digit or subtype) resolution HLA (subtype and haplotype structure frequencies) and CCR5 polymorphism diversity in the individuals that inhabit Honduras, which are non-existent when compared to other countries in the Mesoamerican region and are of direct relevance to HLA-HIV associations and the associated underlying mechanisms. This study also represents the first HLA description to warrant future studies in population genetics, susceptibility, and resistance to other infectious diseases and might also be useful for organ or tissue transplantation (e.g., using haplotype structures for HLA typing quality assurance; estimating probabilities to found donor/receptor pairs) in the country. Additionally, the HLA-pVL/CD4 association outcomes of the present study may be appropriate to design effective HIV vaccines using CTL epitope-based constructs that take into account distinct HLA contexts, like those expressed in the Mesoamerican region.

## Data Availability

HLA haplotype data that support this study are available in supplemental Data Set S1 to be used elsewhere. Likewise, whole raw Sanger sequences of *HLA-A*, *HLA-B*, and *HLA-C* are available in the Dryad repository (Valenzuela-Ponce, Humberto; Ávila-Ríos, Santiago. 2023. HLA class I Sanger sequence data from the Honduras HIV cohort [Dataset]. Dryad https://doi.org/10.5061/dryad.x3ffbg7rc).

## SUPPLEMENTAL MATERIAL

Supplemental material spectrum.01613-23-s0001.xlsx Data Set S1, Tables S1 to S6, and Fig. S1 and S2.

ASM does not own the copyrights to Supplemental Material that may be linked to, or accessed through, an article. The authors have granted ASM a non-exclusive, world-wide license to publish the Supplemental Material files. Please contact the corresponding author directly for reuse.
